# Pregnancy complications in Brazilian puerperal women treated in the public and private health systems ^1^

**DOI:** 10.1590/1518-8345.2156.2949

**Published:** 2018-01-08

**Authors:** Patrícia Louise Rodrigues Varela, Rosana Rosseto de Oliveira, Emiliana Cristina Melo, Thais Aidar de Freitas Mathias

**Affiliations:** 2 PhD, Adjunct Professor, Departamento de Enfermagem, Universidade Estadual do Paraná, Paranavaí, PR, Brazil.; 3 Post-doctoral fellow, Departamento de Enfermagem, Universidade Estadual de Maringá, Maringá, PR, Brazil. Scholarship holder at Coordenação de Aperfeiçoamento de Pessoal de Nível Superior (CAPES), Brazil.; 4 PhD, Adjunct Professor, Departamento de Enfermagem, Universidade Estadual do Norte do Paraná, Bandeirantes, PR, Brazil.; 5 PhD, Full Professor, Departamento de Enfermagem, Universidade Estadual de Maringá, Maringá, PR, Brazil.

**Keywords:** Pregnancy Complications, Pregnancy, Morbidity, Prenatal Care, Healthcare Financing

## Abstract

**Objective:**

to analyze the prevalence of pregnancy complications and sociodemographic profile of puerperal patients with complications, according to the form of financing of the childbirth service.

**Method:**

cross-sectional study with interview of 928 puerperal women whose childbirth was financed by the Unified Health System, health plans and private sources (other sources than the Unified Health System). The sample was calculated based on the births registered in the Information System on Live Births, stratified by hospital and form of financing of the childbirth service. Data were analyzed using the chi-square and Fisher’s exact tests.

**Results:**

the prevalence was 87.8% for all puerperal women, with an average of 2.4 complications per woman. In the case of deliveries covered by the Unified Health System, urinary tract infection (38.2%), anemia (26.0%) and leucorrhea (23.5%) were more frequent. In turn, vaginal bleeding (26.4%), urinary tract infection (23.9%) and leucorrhoea (23.7%) were prevalent in deliveries that were not covered by the Unified Health System. Puerperal women that had their delivery covered by the Unified Health System reported a greater number of intercurrences related to infectious diseases, while women who used health plans and private sources reported intercurrences related to chronic diseases. A higher frequency of puerperal adolescents, non-white women, and women without partner among those assisted in the Unified Health System (p < 0.001).

**Conclusion:**

the high prevalence of complications indicates the need for monitoring and preventing diseases during pregnancy, especially in the case of pregnant women with unfavorable sociodemographic characteristics.

## Introduction

Gestation is a physiological event in the women’s life usually free from complications. However, hundreds of thousands of women die every year due to pregnancy and childbirth complications^1^.

Health problems during pregnancy have increased worldwide, mainly due to complex interactions between demographic factors and lifestyle, as well as advances in modern medicine^2^, with new diagnostic and therapeutic practices.

Among the main clinical pregnancy complications reported in the literature are Urinary Tract Infections (UTIs)^3^ ^-^ ^4^, pregnancy-induced hypertension (PIH), anemia and hyperemesis^5^ ^-^ ^6^. In the United States, a multicenter study of hospitalizations during pregnancy showed a 71% increase in the occurrence of PIH between 1994 and 2011^7^. Another study also carried out in the United States pointed out that the main intercurrences associated with maternal mortality were pre-eclampsia and obstetric hemorrhage^8^.

Another common complaint in pregnancy are urinary tract infections (UTI), with well-known severity and frequency^9^. This complication represents one of the main risk factors for preterm birth and restriction of intrauterine growth, low birth weight and eclampsia^10^. Anemia can occur in up to 19% of pregnant women worldwide according to estimates^11^ ^)^ and it is associated with low sociodemographic profiles, being more common in developing countries^12^.

Women with unfavorable socioeconomic levels, preexisting conditions such as diabetes, hypertension, anemia and heart disease, and adolescents or women over 35 years of age may be more likely to experience undesirable outcomes, since complications during pregnancy are predictors of maternal and fetal morbidity and mortality^13^ ^-^ ^14^. It is estimated that, for each woman who dies during the gestation, another 20 to 30 experience acute or chronic complications, with permanent consequences that impair the body’s functionality^15^. Intercurrences during pregnancy also affect the allocation of financial resources for maternal and child health. In the United States of America, a survey of 137,040 infants between 2007 and 2011 found a prevalence of 75.4% of women with at least one complication during the gestation period, increasing from US $ 987 to US $ 10,287 in the cost of care for newborns^16^.

In Brazil, many programs have been implemented for assistance, prevention and control of morbidity and mortality of women during pregnancy, childbirth and puerperium, especially at the national level, such as the Stork Network*, and state level, such as the Paranaense Mother Network in the State of Paraná^**^. However, these efforts have not fully achieved the expected goals. The Fifth Millennium Development Goal was to achieve a reduction of 75% in maternal mortality rates by 2015, but this was only 45%^1^ worldwide, including in Brazil.

Knowing the prevalence, the main types of diseases and disorders and the sociodemographic characteristics of women with intercurrences during pregnancy may favor the management and prevention of undesirable outcomes for mothers and newborns. Thus, in this study we tried to answer the following questions: what is the prevalence and main types of pregnancy complications? and what is the sociodemographic profile of affected women in a municipality in the Southern Region of Brazil? Population-based studies are necessary, especially those that present a general panorama of intercurrences that, along with the sociodemographic profile, can contribute to the knowledge of health care characteristics and improved care for women in the gestational period. The objective of the present study was to analyze the prevalence of pregnancy complications and the sociodemographic profile of puerperal women with complications, according to the form of financing of the childbirth service.

## Method

This is a cross-sectional study with data from interviews and medical records of puerperal women living in the municipality of Maringá, PR, according to the form of financing of the childbirth service. Maringá is located in the northwest of Paraná, it is the third most populous municipality in the state, had an estimated population of 403,063 inhabitants in 2015, and had a Human Development Index (HDI) of 0.81, the sixth in Paraná^***^.

Childbirth assistance in Maringá is carried out in six hospitals, and two have obstetric beds reserved to the Unified Health System (SUS), one of which is a school hospital and works exclusively for the public health sector. Data was collected in five hospitals, because one did not provide authorization for realization of the research.

The sample was calculated based on 4,656 births of women residing in Maringá, registered in the Information System on Live Births (SINASC) in 2012. Births were stratified by hospital and by type of financing of the childbirth service, either SUS or other sources. In this study, births financed by the Unified Health System are referred as SUS births, and those financed by health plans and individual sources as non-SUS births. The percentages of births per hospital and form of financing of the childbirth service were considered in the study, excluding 638 births that occurred in the private hospital that denied authorization (Table 1). The parameters used for the sample calculation were: alpha error of 0.05, relative frequency of 50% of exposure, and maximum error of estimation of 0.03%. The final figure of 928 puerperal women already includes a 10% increase for possible losses and refusals. The inclusion criteria were: to reside in the municipality of Maringá, with live birth in the analyzed gestation and stay in rooming-in arragement.

**Table 1:** Distribution of births of women residing in Maringá, according to hospital and form of financing of the childbirth service. Maringá, PR, Brazil, 2012

Hospital		N	%	Total	Total+10%
	Hospital 1 (SUS*)	394	9.8	83	91
	Hospital 2 (SUS*)	1727	43.0	362	398
Total SUS		2121	-	445	489
	Hospital 2 (Non-SUS*)	742	18.5	156	172
	Hospital 3 (Non-SUS*)	388	9.7	81	89
	Hospital 4 (Non-SUS*)	498	12.4	105	116
	Hospital 5 (Non-SUS*)	269	6.7	56	62
Total non-SUS*		1897	-	398	439
Total		4018	100	843	928

*Unified Health System

The training for data collection was performed by three nursing doctoral students and a nurse hired for the research and was carried out in meetings with the application of a manual of instructions for the field work. The training also included a pilot test in the hospital that had the greater number of deliveries.

Data were collected from October 2013 to February 2014 in daily visits to the hospitals, including Saturdays, Sundays and holidays, without interruption, until the sample number was completed. After the identification of the puerperal women residing in Maringá through the hospital records, they were approached in the rooming-in and invited to participate in the research. The time assigned for feeding, visits, medical and nursing procedures and care of the newborn were avoided. Only two puerperal women refused to participate in the study, because of wear due to long hospitalization since gestation; these women were replaced. An online form was used in the Google Docs app, which allows agility in both collecting and storing data in spreadsheets. During fieldwork, the spreadsheets were checked daily, aiming at the safety and quality of the data, if necessary further consultation of the medical records and contact with the puerperal women were performed.

For information on the complications, the women were first asked whether they had experienced any of the following problems during pregnancy: vaginal bleeding, syphilis, *Diabetes Mellitus* (DM), gonorrhea/chlamydia, HIV infection, vaginal discharge, Placenta Previa (PP), Premature Membrane Rupture (PMR), polyhydramnios, oligohydramnios, fetal malformation, depression, gestational arterial hypertension and/or Urinary Tract Infection (UTI), with yes/no responses. Then, they were asked: *Did you have any other serious health problems during pregnancy?* If yes, *which problem?* That is, the puerperal women could spontaneously report pregnancy intercurrences without intervention of the interviewer. In order to guarantee greater fidelity of the answers, besides asking whether or not intercurrences had happened, each woman was asked if laboratory tests were performed and if there had been a medical diagnosis and/or treatment for it. The occurrence of leucorrhea was only considered when there was a report of drug treatment. The medical record was consulted to complement other events that were not informed at the interview, as for example: if women did not report polyhydramnios during pregnancy in the interview, but this information existed in the medical record, the later source of information was considered.

The analysis of the prevalence of complications was performed according to the form of financing of the childbirth service, and socioeconomic characteristics, namely: age, ethnicity, area of ​​residence, schooling, economic class (A, B, C, D, E) and prenatal financing, self-reported during the interview. The variable economic class was defined according to the Brazilian Economic Classification Criteria of the Brazilian Association of Research Companies (Abep), which estimates the purchasing power of the persons by the sum of the values ​​of the owned items, plus the sum of the level of education, and establishes eight economic classes. In this study, five classes were considered: strata A1 and A2 (average family income from R$ 9,733.00 to R$ 6,564.00); strata B1 and B2 (average family income from R$ 3,479 to R$ 2,013.00); strata C1 and C2 (average family income from R$ 1,195.00 to R$ 726.00); stratum D (average family income of R$ 485.00) and stratum E (average family income of R$ 277.00). The financing of prenatal care indicates if the prenatal care was performed in the public (SUS) or private (non - SUS) health system or both (mixed).

Data were described by means of absolute and relative frequencies and the comparison between the proportions was carried out by the chi-square test and the Fisher's exact test, when necessary. Interviews and consultation of medical records were carried out after contact with the puerperae, explanation of the objectives of the study and signing of the Informed Consent Term (ICT). The study was approved by the Permanent Committee of Ethics in Research of the State University of Maringá (UEM), PR (nº 800,748/2014).

## Results

Eight hundred and fifteen (87.8%) out of 928 puerperal women reported at least one complication during pregnancy, with an average of 2.4 complications per woman, 2.5 for those who had SUS births and 2.4 for those who had non-SUS births. Only 14.7% of puerperal women who had SUS births and 9.3% with non-SUS births did not have complications during pregnancy (p = 0.012). Women who had non-SUS births reported more complications than those who had SUS births, both in the case of one (21.2 and 19%, respectively) and for two (20.5 and 14.9%, respectively) complications, (p = 0.026). The data in question are described in the Table 2.

**Table 2:** Frequency of complications (n and %), according to the form of financing of the childbirth service. Maringá, PR, Brazil, 2013-2014

Complications	SUS*			Non-SUS*			Total		p
	n	%		n	%		n	%	
None	72	14,7		41	9,3		113	12,2	0,012
1	93	19,0		93	21,2		186	20,0	0,411
2	73	14,9		90	20,5		163	17,6	0,026
3	81	16,6		72	16,4		153	16,5	0,947
4 or more	170	34,8		143	32,6		313	33,7	0,481
Total	489	100,0		439	100,0		928	100,0	

*Unified Health System

UTI (31.5%), followed by anemia (24.4%), leucorrhea (23.6%), vaginal bleeding (23.5%), Preterm Labor (PTL) (22.6%) and PIH (19.5%) were the most frequent complications. A high frequency of UTI (p < 0.001) and oligohydramnios (p = 0.013) was observed among puerperal women who had SUS births, and a high frequency of PMR (p < 0.001), gestational diabetes (p=0.042), PP (p < 0.001) and polyhydramnios (p < 0.001) among those who had non-SUS births. With fewer cases (less than 2%), syphilis, gonorrhea, HIV infection, condylomata, HPV, rubella and hepatitis B occurred more frequently in the pregnancy of puerperal women who had SUS births (Table 3).

**Table 3:** Distribution of complications, according to the form of financing of the childbirth service. Maringá, PR, Brazil, 2013-2014

Complications‡	SUS*			Non-SUS*			Total^†^		p
	n	%		n	%		n	%	
Urinary tract infection	187	38,2		105	23,9		292	31,5	<0,001
Anemia	127	26,0		99	22,6		226	24,4	0,226
Leucorrhea	115	23,5		104	23,7		219	23,6	0,951
Vaginal bleeding	102	20,9		116	26,4		218	23,5	0,046
Preterm labor	107	21,9		103	23,5		210	22,6	0,566
PIH^§^	106	21,7		75	17,1		181	19,5	0,078
PA^||^	32	6,5		80	18,2		112	12,1	<0,001
Gestational diabetes	33	6,7		46	10,5		79	8,5	0,042
Oligohydramnios	48	9,8		24	5,5		72	7,8	0,013
Placenta previa	9	1,8		38	8,7		47	5,1	<0,001
Depression	23	4,7		14	3,2		37	4,0	0,239
Polyhydramnios	8	1,6		28	6,4		36	3,9	<0,001
PMR^¶^	20	4,1		8	1,8		28	3,0	0,044
Renal calculus	12	2,5		8	1,8		20	2,2	0,508
Hypothyroidism	7	1,4		10	2,3		17	1,8	0,337
Syphilis	7	1,4		1	0,2		8	0,9	0,072**
Hyperemesis	2	0,4		4	0,9		6	0,6	0,430**
Intrauterine infection	2	0,4		3	0,7		5	0,5	0,672**
Gonorrhea	5	1,0		-	-		5	0,5	
*Influenza A*	1	0,2		4	0,9		5	0,5	0,195**
Nausea	-	-		4	0,9		4	0,4	
Toxoplasmosis	3	0,6		1	0,2		4	0,4	0,626**
Vaginal herpes	1	0,2		2	0,5		3	0,3	0,605**
HIV^††^	2	0,4		-	-		2	0,2	
Condyloma	2	0,4		-	-		2	0,2	
Hypoglycemia	1	0,2		1	0,2		2	0,2	1**
*Human papilloma virus*	2	0,4		-	-		2	0,2	
Fetal growth restriction	-	-		2	0,5		2	0,2	
Rubella	2	0,4		-	-		2	0,2	
Cytomegalovirus	-	-		1	0,2		1	0,1	
Dengue	-	-		1	0,2		1	0,1	
Dyspnea	-	-		1	0,2		1	0,1	
Gestational sac hematoma	1	0,2		-	-		1	0,1	
Hepatitis B	1	0,2		-	-		1	0,1	
Infection of the uterine cervix	1	0,2		-	-		1	0,1	
String failure	1	0,2		-	-		1	0,1	
Venous lake	-	-		1	0,2		1	0,1	
Positive Papanicolaou	1	0,2		-	-		1	0,1	
Blood incompatibility	1	0,2		-	-		1	0,1	
Ovarian bleeding	-	-		1	0,2		1	0,1	
*Streptococcus vaginalis*	1	0,2		-	-		1	0,1	
Hyperglycemia	1	0,2		-	-		1	0,1	
Intrauterine infection	1	0,2		-	-		1	0,1	
Others‡‡	41	8,4		64	14,6		105	11,3	0,003
Total	1016	-		949	-		1965	-	-

*Unified Health System; † Each pregnant woman could give more than one response ; ‡The puerperae were asked and could give yes/no answers for each complication; §Pregnancy-Induced Hypertension; ||Premature Placental Abruption; ¶Premature Membrane Rupture; **Fisher exact test; ††Human Immunodeficiency Virus; ‡‡Complications reported by the puerperae in response to the question: Did you have any other serious health problems during pregnancy? In this category, 51 health problems were cited

Among sociodemographic characteristics, there were differences according to the source of financing of the childbirth service, showing vulnerability in the case of puerperal women who had SUS births. The percentage of adolescents with complications was higher among puerperal women who had SUS births (16.1%) than among those who had non-SUS births (9.1%) (p < 0.001), as well as the percentage for the non-white ethnicity (57.8% of the SUS births and 32.4% of the non-SUS births, p < 0.001); less than 8 years of schooling (13.2% of SUS births and 2.3% in non-SUS births, p < 0.001) and no partner (14.4% of the SUS births and 2.8% of the non-SUS births; p < 0.001). Regarding the economic class, 56.8% of puerperal women who had SUS births and 17.7% of those who had non-SUS births were classified as class C; and 35.7% of puerperal women who had SUS births and 73.1% of those who had non-SUS births were classified as class B (p < 0.001). As expected, the majority of women who had SUS births (91.6%) received prenatal care exclusively in the public network, whereas the majority of puerperal women with non-SUS births (99.5%) had prenatal care in the private or mixed network (Table 4).

**Table 4:** Sociodemographic characteristics of puerperae with pregnancy complications, according to the form of financing of the childbirth service. Maringá, PR, Brazil, 2013-2014

		SUS* (417)			Non-SUS* (398)			Total (815)		p
		n	%		n	%		n	%	
Age										
	<20	67	16,1		8	2,0		75	9,2	<0,001^†^
	20 to 34	300	71,9		323	81,2		623	76,4	0,357
	35 or more	50	12,0		67	16,8		117	14,4	0,116
Ethnic group										
	White	176	42,2		269	67,6		445	54,6	<0,001
	Non-white	241	57,8		129	32,4		370	45,4	<0,001
Residence zone										
	Urban	408	97,8		396	99,5		804	98,6	0,672
	Rural	9	2,2		2	0,5		11	1,35	0,035^†^
Schooling										
	< 8 years	55	13,2		9	2,3		64	7,8	<0,001^†^
	≥ 8 years	362	86,8		389	97,7		751	92,1	0,325
Partner										
	Yes	357	85,6		387	97,2		744	91,3	0,271
	No	60	14,4		11	2,8		71	8,71	<0,001
Economic class^‡^										
	A	4	1		37	9,3		41	5,0	<0,001^†^
	B	149	35,7		291	73,1		440	54,0	<0,001
	C	237	56,8		70	17,6		307	37,7	<0,001
	D	22	5,3		-	-		22	2,7	-
	E	5	1,2		-	-		5	0,6	-
Prenatal financing										
	SUS	382	91,6		2	0,5		384	47,1	<0,001
	Non-SUS* or mixed	35	8,4		396	99,5		431	52,9	

* Unified Health System; †Fisher’s exact test; ‡ Brazilian Economic Classification Criteria of the Brazilian Association of Research Companies (Abep)

## Discussion

The high prevalence of pregnancy complications reported by the puerperae of Maringá, in the case of both, women whose childbirth was financed by the public or the private sector, is important in this study. Some complications and aggravations during pregnancy are, to some extent, expected, because imbalances of the metabolic, circulatory, neurological and renal functions may occur in the gestational period, when the physiological woman’s balance is quite altered. However, the frequency in this study was considerably high when compared to results found in a cohort study conducted in Missouri, United States, where a frequency of 32.3% was found, despite taking into account the differences in the socioeconomic profile of the population and in the form of data collection^17^.

In addition to the high prevalence of pregnancy complications of women living in Maringá, for both types of childbirth financing, some aspects show the vulnerability of SUS users, such as a higher percentage of women with four or more intercurrences even, although without significant association. This disadvantage should be considered mainly by guaranteeing the access and quality of public health services and also by observing the sociodemographic characteristics of the SUS user population associated with the prevalence of complications.

The main complications reported by the puerperae, such as UTI, anemia, leucorrhea, vaginal bleeding, PTL and PIH, are in agreement with the literature^4^ ^,^ ^6^, but with different prevalence values. The most reported complication during pregnancy was higher than the expected mean, which is 20%*, the same as in a study conducted in Nigeria in 2011, in which a prevalence of 21% was found^18^.

Responsible for approximately 10% of antepartum hospitalizations, UTIs, sometimes asymptomatic, may progress to pyelonephritis and cystitis and trigger complications for the fetus, such as premature birth and low birth weight^9^ ^,^ ^15^. These complications can be avoided by care quality during pregnancy, diagnosis and early treatment, as recommended by national, state and municipal protocols for prenatal care^19^.

Anemia, unrelated to the form of financing of the childbirth, was below the expectation compared to the World Health Organization (WHO) estimates for developing countries, which ranges from 40 to 59.9%^20^. A study carried out in a hospital in Ethiopia in 2012 to analyze the prevalence and predictors of maternal anemia, there was a prevalence of 16.6% of anemia^21^, a lower value than that found in the present study. As a public health problem affecting low, middle and high income countries, the effects of anemia during pregnancy include low birth weight, some neurological diseases of the fetus and increased risk of maternal and perinatal mortality^20^.

Vaginal bleeding was reported by the interviewed puerperae, with a higher proportion of those with childbirths financed by health plans or private sources (non-SUS). Bleeding during pregnancy has been associated with PMR and the placenta previa*. In this study, PMR and placenta previa* were observed most often in women who had non-SUS births.

Although these results show a higher frequency of vaginal bleeding among women who had non-SUS births, it was also reported by women who had SUS births. This occurrence may be related to the ease of detection, without the need for clinical or laboratory tests. On the other hand, the higher prevalence of PMR and placenta previa among puerperal women who used health plans or private sources (non-SUS) may indicate more information and faster access to health care, with diagnostic tests and explanations that led them to report these occurrences. PMR occurs more frequently during the 24th to 26th week of pregnancy, and less frequently in the subsequent weeks^22^, and consists of the separation of the placenta implanted in the uterus^23^. In the present study, the highest frequency of PMR among women who had non-SUS births may be associated with the highest frequency ages above 35 years, a major risk factor for PMR* in this group.

PIH presented a higher incidence in this study than that found in a review with pregnant women from several countries of the world, which reported a incidence of 5.2 to 8.2%^24^. In this study, it was observed that the highest prevalence of PIH occurred among puerperal women who had non-SUS births, and the highest prevalence of gestational diabetes among those who had SUS births. This is interesting, because the risk factors for PIH and gestational diabetes are similar, including age over 35 years, sedentary lifestyle, obesity, inadequate diet, hormonal factors and use of continuous medications, as well as the possible complications resulting from PIH and gestational diabetes, which include polyhydramnios, eclampsia, PTL, congenital malformation, maternal and infant mortality, among others. Age over 35 years and black and brown ethnicity are demographic characteristics identified as determinants for PIH, which is one of the complications with incidence of 5 to 10% in pregnant women*. PIH is considered a major cause of maternal morbidity and mortality in developing countries, with high rates of severe maternal morbidity and maternal mortality in Brazil^25^.

Other diseases reported in this study had a higher percentage among women who had SUS births, with also higher proportion of some infectious diseases such as Hepatitis B, syphilis, gonorrhea, toxoplasmosis, HIV, Condyloma, HPV and rubella. From 26 cases of these diseases, 24 occurred in the group of women assisted by the SUS. Syphilis was the subject of a multinational study in 2008, in which approximately 1,360,485 women with syphilis were found, mainly in Africa and South America, in populations with characteristics of higher vulnerability, namely adolescents, with low schooling and without partner^26^. On the other hand, in the present study, a higher proportion of specific gestational diseases or chronic diseases were observed in women who had SUS births. This result may be associated with maternal age, as a higher proportion of puerperae were aged 35 years or more in this group, ages when chronic non-transmissible diseases and gestational risks are more prevalent.

## Conclusion

There are some limitations in this study, including the use of self-reported information, what incurs the possibility of memory bias of puerperal women. However, the collection of information about complications in the interview, with two questions, one prompting stimulated and the other spontaneous answers, proved to be a viable strategy, because a high prevalence and diversity of problems were reported and suggest that the puerperae still remember these occurrences during pregnancy. The fact that the severity of the complications was not classified and that the financing of the childbirth service categorized as non-SUS included all private health plans, without differentiation of the types of coverage, which are known to be variable, should also be considered as a limitation.

Some relevant aspects related to the health of women in a medium-sized municipality were highlighted in this study, driving attention to the high prevalence of complications during pregnancy and the differences in the socioeconomic profile of puerperal women according to the form of financing of the childbirth service. A higher percentage of adolescents, non-white ethnicity, women without partner, and women belonging to unfavorable economic class, were observed among the puerperal women who had childbirth financed by the public health sector. These results show the need to improve prenatal care, with appropriate actions to prevent and monitor pregnancy complications. Health teams should be prepared to prevent and treat major events as early as possible and, in particular, identify those with the potential to trigger more serious complications, especially if they occur in women in situations of vulnerability, both social and biological.

## Footnotes

* Montenegro CAB, Rezende Filho J. Rezende fundamental obstetrics. 12. ed. Rio de Janeiro: Guanabara Koogan; 2011
